# Hydroxyurea and colonic ulcers: a case report

**DOI:** 10.1186/1471-230X-14-134

**Published:** 2014-07-31

**Authors:** Kochawan Boonyawat, Sansanee Wongwaisayawan, Prawat Nitiyanant, Vichai Atichartakarn

**Affiliations:** 1 Division of Hematology, Department of Medicine, Faculty of Medicine Ramathibodi Hospital, Mahidol University, Bangkok, Thailand; 2 Department of Pathology, Faculty of Medicine Ramathibodi Hospital, Mahidol University, Bangkok, Thailand

**Keywords:** Hydroxyurea, Colonic and oral ulcers, Thalassemia

## Abstract

**Background:**

Hydroxyurea at a relatively low dose is frequently prescribed to induce hemoglobin F production in patients with sickle cell and β-thalassemia diseases because of its good efficacy and safety profiles. However, a potentially fatal gastrointestinal ulceration was recently found and herein reported.

**Case presentation:**

A thirty-seven-year-old man with transfusion dependent hemoglobin E/β-thalassemia disease was treated with hydroxyurea to induce hemoglobin F production since 2007 without incident. From 2008 to April 2010, episodes of hematochezia, mucous diarrhea and epigastric pain intermittently manifested. Four colonoscopies done during the period repeatedly showed ulcerative lesions from the terminal ileum to the ascending colon with a non-specific histo-pathologic finding. Subsequently, ulcerative lesions also developed at the pharynx, histo-pathologic findings of which were not different from those in the colon. These ulcerative lesions resolved within a month after discontinuing hydroxyurea in April 2010 and have not recurred since.

**Conclusion:**

The findings suggested role of hydroxyurea in the pathogenesis of these ulcers, and that it must be immediately discontinued to prevent further damage to the digestive mucosa.

## Background

Hydroxyurea is an anti-metabolite being prescribed in many hematologic disorders. It acts by inactivating the ribonucleotide reductase enzyme leading to the inhibition of DNA synthesis. Its main therapeutic use is in chronic myeloproliferative neoplasm. However, in view of its ability to induce hemoglobin (Hb) F production, it has also been used in patients with sickle cell and β-thalassemia diseases [1,2]. The most common side effect of hydroxyurea is myelosuppression. Other common side effects are gastrointestinal (GI), such as nausea, vomiting, diarrhea, anorexia and stomatitis, as well as cutaneous, such as skin hyperpigmentation, nail changes and alopecia [3,4]. Unusual mucocutaneous side effects are leg and rarely oral ulcers, which may occur after a prolonged use, and are reversible after drug cessation [5-8]. Leukocytoclastic vasculitis was shown in biopsies of leg ulcers from patients with chronic myeloproliferative neoplasm who had been on this drug [9-11]. Hydroxyurea-related GI ulcer is extremely rare. A patient with myelofibrosis was reported to have bled from jejunal ulcers, which showed features compatible with hypersensitivity vasculitis [12]. Herein, we report another unusual side effect, which is potentially fatal, to raise its awareness so that proper management can be done early.

## Case presentation

A thirty-seven-year-old Thai male was diagnosed with hemoglobin E/β-thalassemia (E/β-Thal) disease at four years of age. Splenectomy was done in December 2000 (23 years old) because of increasing blood transfusion requirement. However, 2 units of packed red blood cells transfusion were still required monthly to relieve anemic symptom. Pulmonary hypertension was diagnosed in February 2004. Hydroxyurea at 500 mg/d five days a week was prescribed in November 2007 to see if it could improve symptoms and transfusion requirement. Other medications were warfarin, aspirin, deferoxamine and folic acid. Baseline Hb was 7 g/dL.

In December 2007, acute massive hematochezia after naproxen usage in the preceding 2 weeks for plantar fasciitis necessitated hospital admission. Physical examination showed a markedly pale man with moderately icteric sclerae. Height and weight were 174 cm and 54 kg, respectively. The precordial area was mild to moderately active with increased P_2_. Liver edge was palpable 3 cm below the right costal margin. Prothrombin time international normalized ratio was 1.8. Blood transfusion followed by colonoscopy was prescribed. The latter showed multiple shallow ulcers at the cecum and normal-looking ileo-cecal (IC) valve. Histo-pathology of the biopsied specimen showed acute ulcers with chronic inflammation and severe tissue eosinophilia. Although the findings were non-specific, naproxen was discontinued. Periodic epigastric pain and mucous diarrhea persisted and were not alleviated by antacid or proton pump inhibitor. Meanwhile, hydroxyurea dosage was increased to 4.5 g/week due to a poor response.

Follow-up colonoscopy a year later showed an ulcerated IC valve with multiple small ulcers along the terminal ileum to the ascending colon. The differential diagnosis from endoscopic findings was infection, such as tuberculosis, cytomegalovirus, as well as non-infectious causes, such as Crohn’s disease and malignancy. Ischemic bowel disease was less likely because of his young age and no sharp demarcation of lesion according to the territorial vascular blood supply. Biopsies of the ulcers showed acute and chronic inflammation with tissue eosinophilia which was non-specific. There was no thrombus in the blood vessels. Special stain for acid fast bacilli (AFB) and Gomori methenamine silver (GMS) showed no organisms. Polymerase chain reaction for mycobacterium tuberculosis was negative. Thus, no specific treatment was given. Periodic epigastric pain and occasional mucous diarrhea persisted.

**Figure 1:**
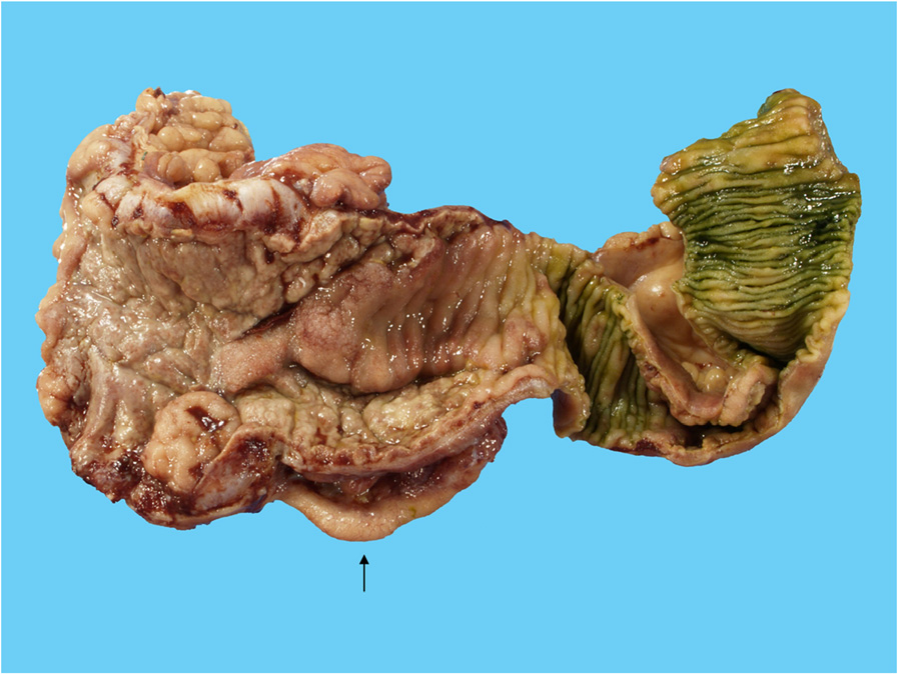
**Gross pathologic findings of the terminal ileum, the ileo-cecal valve and the cecum (from right to left).** The terminal ileum shows acute ulcers with elevated ulcer rim and irregular base. The mucosa of the ileo-cecal valve and the cecum shows a confluent ulcer with an elevated border. The submucosa is markedly edematous. The vermiform appendix (↑) is mildly congested.

**Figure 2:**
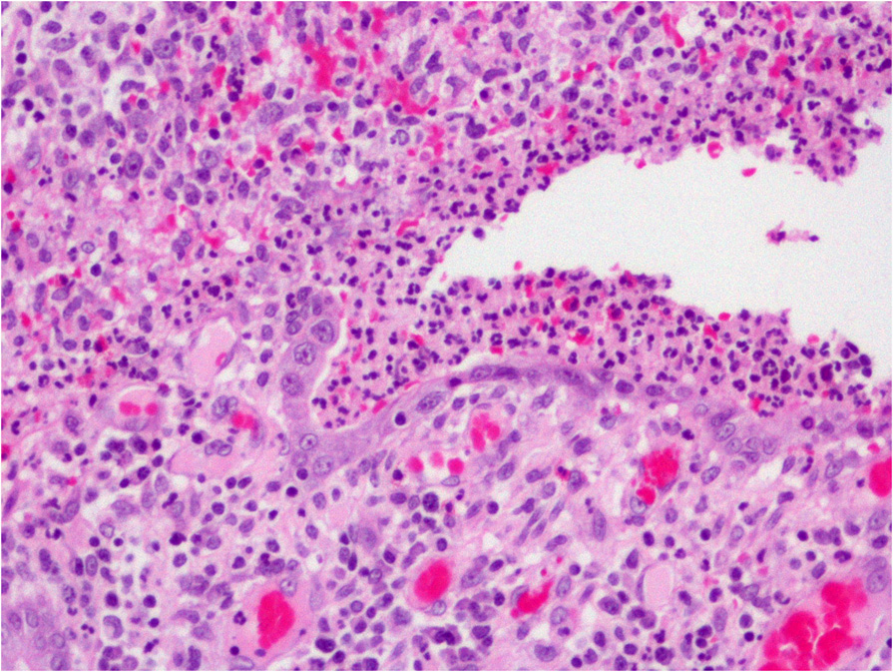
**Histo-pathologic finding of the ascending colon (H&E × 200).** The mucosa shows acute and chronic inflammatory cells infiltration with ulceration.

In December 2009, he presented with fever and mucous diarrhea. CT scan of the whole abdomen showed a circumferential thickened wall involving the ascending colon and the terminal ileum. Esophago-gastro-duodenoscopy revealed multiple erosions on the gastric mucosa, biopsy of which showed chronic gastritis. Colonoscopy revealed a markedly swollen and ulcerated IC valve and circumferentially thickened wall from the terminal ileum to the ascending colon. Biopsies of cecal ulcers showed acute ulcers with acute and chronic inflammatory cells infiltration to the muscularis mucosae. There was no feature of malignancy, vasculitis, viral inclusion or granuloma. GMS, Periodic Acid Schiff (PAS), and AFB stains were non-revealing. Various serologic tests for systemic vasculitis, such as antinuclear antibody, perinuclear and cytoplasmic antineutrophil cytoplasmic autoantibody were all negative. Empiric treatment with intravenous ceftriaxone was given.In February 2010, there was another episode of massive hematochezia, resulting in a partial right half colectomy. Multiple scattered ulcerative lesions involving the terminal ileum, the cecum and the ascending colon were found on gross examination (Figure 1). The histo-pathologic findings were acute mucosal ulcers with granulation tissue and tissue eosinophilia. The submucosa was thickened and edematous. The acute and chronic inflammatory cells infiltrated through the submucosa to the inner muscular layer, not unlike the previous ones (Figure 2). Leukocytoclastic vasculitis was not found. Special stains with GMS, PAS, and AFB were again non-revealing.

A month later, odynophagia was complained. Fiberoptic laryngoscopy showed a whitish ulcerative plaque at the right tonsillar fossa extending to the right posterior pharyngeal wall and the left tonsillar fossa. Punch biopsy was done. Symptoms got worse with lesions progressing to the hard palate, the pyriform sinus and the posterior pharyngeal wall. Histo-pathologic findings showed acute and chronic inflammatory changes with granulation tissue and tissue eosinophilia, not unlike those in the colon.

Hydroxyurea was discontinued in April 2010 because drug-induced ulceration was suspected. Odynophagia markedly improved. Two month-follow-up fiberoptic laryngoscopy showed clearing of all previous lesions. All GI symptoms disappeared without recurrence to date.

### Discussion

Ulcerative oral lesion is a rare adverse effect of hydroxyurea. All reported cases used hydroxyurea for chronic myeloproliferative neoplasm [5-8]. The lesions were confined to the lips, base of tongue and buccal mucosa, and were not related to myelosuppression or cumulative dose. They completely healed shortly after drug cessation. In some patients, the ulcers recurred after drug re-administration, reaffirming its causative role.

Our patient’s GI symptoms started about a month after having been on a low dose hydroxyurea for Hb F induction. A history of massive hematochezia following naproxen usage in the preceding 2 weeks led us to believe that naproxen was the cause. However, persistence of occasional mild epigastric pain together with a small amount of mucous diarrhea with and without blood despite naproxen cessation, and additional treatment with several proton pump inhibitors made it less likely. Due to a poor hematologic response, the hydroxyurea dose was increased. Abdominal symptoms remained the same despite the mildly progressive ulcerative lesions shown in the two consecutive annual colonoscopies. Shortly afterwards, a massive hematochezia developed, necessitating a partial right half colectomy. A more extensive investigation of the specimen for evidence of infectious causes, such as mycobacterium tuberculosis and cytomegalovirus, and for non-infectious causes, such as malignancy, inflammatory bowel disease and systemic vasculitis were non-revealing. Leukocytoclastic and hypersensitivity vasculitis were not observed in the ulcerative area. Thus, there was no change in the treatment plan.

Development of new painful oral ulcers, histo-pathologic findings of which were not unlike those in the colon led us to suspect that etiology of ulcers at these various sites was the same. Hydroxyurea was suspected because it can, on rare occasions, induce oral ulcers. After its cessation, odynophagia markedly improved in a month, and all GI symptoms disappeared in 2 months. Without recurrence up to now (4 years), the finding strongly suggested hydroxyurea as the culprit.

Hydroxyurea-related ulcers were previously reported to be confined to the oral area, the pathogenesis of which was unknown. Oral ulcers are not uncommon after methotrexate, also an anti-metabolite, and are related to the dose and severity of myelosuppression [13]. However, there was no myelosuppression in the reported cases of hydroxyurea-related oral ulcers. The dose of hydroxyurea used in our patient was relatively low, and there has never been myelosuppression. Thus, pathogenesis of these ulcers based on drug’s action on rapidly dividing cells is unlikely. Ours is the first report to show that these ulcers could also occur in the lower part of the GI tract. Its confinement to the ileum and the right-sided colon is intriguing and of unknown cause. Tuberculosis and amoebiasis also have a predilection for this region. The process of ulceration seemed to be very slowly progressive and reversible after drug cessation.

Hydroxyurea is more widely used in sickle cell than in β-thalassemia disease, and there never have been a report of ulcers such as those seen in our patient [14]. Taken together, these ulcers are likely to be idiosyncratic.

In contrast to hydroxyurea, non-steroidal anti-inflammatory drug-induced ulcers are typically confined to the stomach and small intestine [15]. Histo-pathologic findings of these ulcers, however, are not different, showing a non-specific infiltration by both eosinophils and neutrophils during the acute process. In addition to drug hypersensitivity, eosinophilic infiltration could also be due to allergy, vasculitis and parasitic infestation.

## Conclusion

A very rare adverse effect of hydroxyurea on the GI mucosa in a splenectomized patient with E/β-Thal disease is reported to raise awareness of this potentially fatal but reversible disease. It can be managed simply by drug discontinuation.

### Consent

Written informed consent was obtained from the patient for publication of this case report and any accompanying images. A copy of the written consent is available for review by the Editor of this journal.

## Competing interests

The authors declare that they have no competing interest.

## Authors’ contribution

KB drafted the manuscript; SW and PN made the pathological diagnosis; VA attended the patient and revised the manuscript. All authors read and approved the final manuscript.

## Pre-publication history

The pre-publication history for this paper can be accessed here:

http://www.biomedcentral.com/1471-230X/14/134/prepub
